# Healthcare professionals’ experiences and thoughts on eating and drinking with acknowledged risks in older adults: a comparison of Japan and the UK

**DOI:** 10.1093/ageing/afaf380

**Published:** 2026-01-22

**Authors:** Yuki Yoshimatsu, Marianne Markowski, David Graeme Smithard, Dharinee Hansjee, Tadayuki Hashimoto, Hiroyuki Nagano, Ryan Essex

**Affiliations:** Guy’s and St Thomas’ Hospitals NHS Foundation Trust, Ageing and Health, London, England, United Kingdom of Great Britain and Northern Ireland; Lewisham and Greenwich NHS Trust, Elderly Care, Queen Elizabeth Hospital, London, England, United Kingdom of Great Britain and Northern Ireland; University of Greenwich, The Institute for Lifecourse Development, Centre for Exercise Activity and Rehabilitation, School of Human Sciences, London, England, United Kingdom of Great Britain and Northern Ireland; University of Greenwich, The Institute for Lifecourse Development, Centre for Chronic Illness and Ageing, School of Human Sciences, London, England, United Kingdom of Great Britain and Northern Ireland; University of Greenwich, The Institute for Lifecourse Development, Centre for Professional Workforce Development, London, England, United Kingdom of Great Britain and Northern Ireland; Lewisham and Greenwich NHS Trust, Elderly Care, Queen Elizabeth Hospital, London, England, United Kingdom of Great Britain and Northern Ireland; University of Greenwich, Centre for Exercise Activity and Rehabilitation, School of Human Sciences, London, England, United Kingdom of Great Britain and Northern Ireland; University of Greenwich, Speech and Language Therapy, School of Health Sciences, London, England, United Kingdom of Great Britain and Northern Ireland; Osaka Medical and Pharmaceutical University, Department of General Medicine, Takatsuki, Osaka, Japan; Brigham and Women's Hospital, Department of Emergency Medicine, Boston, Massachusetts, USA; Kyoto University, Department of Healthcare Economics and Quality Management, Graduate School of Medicine, Kyoto, Kyoto Prefecture, Japan; Tenri Yorozu Hospital, General Medicine, Tenri, Nara, Japan; University of Greenwich, The Institute for Lifecourse Development, Centre for Chronic Illness and Ageing, School of Human Sciences, London, England, United Kingdom of Great Britain and Northern Ireland; University of Greenwich, The Institute for Lifecourse Development, Centre for Professional Workforce Development, London, England, United Kingdom of Great Britain and Northern Ireland; The George Institute, Injury Division, Sydney, New South Wales, Australia; University of New South Wales, School of Population Health, Sydney, New South Wales, Australia

**Keywords:** choking, comfort feeding, modified diet, deglutition disorders, pneumonia, aspiration, older people

## Abstract

**Purpose:**

Older adults are commonly restricted of oral intake due to concerns of aspiration. Eating and drinking with acknowledged risks (EDAR) is an alternative pathway that facilitates comfort, dignity and autonomy. However, EDAR decision-making is difficult, with guidance only existing in the UK, and support not readily available. This study was the third in a mixed-methods project aiming to understand how to develop EDAR further whilst providing clinicians with optimal support. This study aimed to reveal the factors that shape confidence in healthcare professionals regarding EDAR decision-making.

**Methods:**

We performed a survey regarding the experiences of healthcare professionals in Japan and the UK with EDAR in older adults. We developed the survey based on themes extracted from our previous qualitative study.

**Results:**

There were 1452 responses (1058 Japanese, 394 UK). Confidence towards EDAR was higher in UK-based respondents (β = 2.358, SE = 0.137, *P* < .001), with greater years of experience (β = 0.028 per year, SE = 0.005, *P* < .001), higher rate of clinical work related to EDAR (β = 0.341, SE = 0.022, *P* < .001), a more involved role in the decision-making, and being clinicians who are not doctors. Similar results were observed regarding likeliness to support EDAR, likeliness to acknowledge perceived benefits, and lower levels of difficulty in undertaking EDAR. Framework (such as guidelines/protocols) and education were ranked to be most beneficial in both countries.

**Conclusions:**

Confidence towards EDAR-decision making was shaped through multiple internal and external factors. Acknowledging these allows us to identify areas in need and provide culturally adapted support, leading to improved experiences in patients, families and healthcare professionals.

## Key Points

- Eating and drinking with acknowledged risks (EDAR) is important for those who prefer to eat and drink despite risk of aspiration.
- Healthcare professionals' confidence in supporting EDAR decision-making is shaped through multiple factors.
- To build confidence, framework such as guidelines alone are not sufficient, and education, training and support are essential.

## Introduction

Eating and drinking are basic human rights which many people enjoy. However, it is commonly denied in patients with suspected dysphagia, due to a feared risk of medical decisions ‘causing’ aspiration or choking. This is evident in the high rate of older patients being placed nil by mouth (NBM) or modified diets on admission for pneumonia [1–3], despite the efficacy of diet modification lacking evidence [4].

Eating and drinking with acknowledged risks (EDAR) is an alternative pathway that facilitates comfort, dignity, and autonomy for patients who prefer to continue oral intake despite there being a risk of aspiration or choking [5]. EDAR can be applied to patients of all ages and disease stages, however, its application appears limited to older adults and in end-of-life settings [2, 6]. This may be attributed to a number of factors including the focus of medicine being curative than comfort and behaviours of healthcare professionals towards the negative health consequences of EDAR such as aspiration, dehydration and malnutrition.

EDAR has been trialled across the world, however, the UK is the first country to have national guidance published in 2021, by the Royal College of Speech and Language Therapists (RCSLT) [5]. The Royal College of Physicians also have a document on supporting eating and drinking in end-of-life care [7].

A global search on EDAR reveals a visual scenario on EDAR made by the Australian Government [8] whilst Speech Pathology Australia have issued a position statement regarding risk feeding [9], although these sources are not as complete as the RCSLT guidance in regards to the decision-making process. In Canada, a framework was developed for patients who decline diet modification [10]. In South Africa, there is a call for guidance in this area of care but nothing formal in place currently [11]. In Japan, although there is acknowledgement of the concept of EDAR [6], there has been no formal guidance towards EDAR decision-making at a national level. The presence or lack of guidance is expected to have an impact on healthcare professionals’ confidence in supporting decision-making around eating and drinking, along with other cultural and systemic differences.

Confidence of the practitioner is a crucial contributor to decision-making, particularly in EDAR, requiring an acknowledgement of the risks involved. Confidence is a multifactorial concept, based on internal and individual factors of the practitioner and external factors in the environment [12–17]. In this research we explored the factors that support confidence in the decision-making for EDAR.

The UK guidance recommends that, the decision-making process of EDAR include a clinical evaluation of the swallow, mental capacity assessment, establishing the goal of care, facilitating communication within the multidisciplinary team, and setting out an advance care plan where appropriate [5]. Other factors to consider include staff training, accurate documentation, review of medication, and appropriate monitoring depending on the setting [18]. These steps are crucial in the implementation of a complex pathway [19].

With the implementation of EDAR in Japan where there is no guidance, and the UK where there is relatively new guidance in place, we formed a unique team of clinicians and academics in both countries and planned a three-phase mixed-methods project on EDAR. The value of this study lies in the comparison of Japan and the UK, both ageing societies with a public healthcare system and national organisations governing dysphagia but with differing support towards EDAR. This project aimed to understand how to provide clinicians with optimal support towards confidence in EDAR decision-making and application whilst staying culturally sensitive. The project employed an exploratory sequential mixed-method design looking at: (i) how EDAR is implemented [2], (ii) what factors are involved in the decision-making and how these differ between the countries [6], and now in the third phase, a snapshot of the state of adoption of EDAR by the wider healthcare profession and their confidence in EDAR decision-making.

In this paper, we detail the third phase of this project, a quantitative survey study on the application of EDAR and views of a wider range of healthcare professionals involved in EDAR decision-making in older adults. The aim of this study was to reveal the factors that shape confidence in healthcare professionals for EDAR decision-making as well as the barriers and facilitators, through comparison of those working in Japan and the UK. Our results are expected to lead to improved guidance and support on EDAR.

## Methods

### Study design

We performed a survey regarding the experiences of healthcare professionals with EDAR in older adults. This paper follows the Consensus-Based Checklist for Reporting of Survey Studies (Supplementary Appendix 1) [20].

As EDAR is novel and there is little published evidence, we developed our survey (Supplementary Appendix 2) based on themes extracted from our qualitative research, which explored the barriers and facilitators in decision-making around EDAR through interviews in healthcare professionals [6]. This exploratory sequential study design [21] was chosen to base the survey on the most recent evidence and maximise the range of possible answers, whilst minimising bias. We developed the survey using the Qualtrics software (Qualtrics, Provo, UT). A bilingual researcher translated the survey into Japanese, and two other bilingual researchers independently checked the translation for accuracy. We piloted the survey with 10 healthcare professionals (constituting of five or more professions each) in each country to conduct multiple trial runs and refined the survey according to their feedback.

### Survey distribution

The survey was distributed through the contacts of researchers and their colleagues and through relevant professional bodies including, in the UK: the RCSLT, the British Geriatric Society and Hospice UK, and in Japan: the Japan Society of Logopedics and Phoniatrics, the Japanese Association of Communication Disorders, the Japanese Association of Speech-Language-Hearing Therapists and Japan Primary Care Association. The survey was open from 15th December 2023 to 30th April 2024.

### Participants

Healthcare professionals and care home workers aged 18 years-old or above, working in the UK or Japan and who have professional experience of caring for older adults with dysphagia were eligible to participate. Participants who took part in the qualitative part of this project were excluded, as their experiences were already extracted through the interviews. Ethical approval was obtained from the Ethics Committee of Hashimoto Municipal Hospital (ID R2.10–1) in Japan, and the Health Research Authority and Health and Care Research Wales (ID 321158) in the UK. Informed consent was obtained from all participants prior to participation. All answers were anonymised and no traceable data were recorded.

### Survey design

We designed the survey to be composed of four sections, mainly collecting quantitative data, with some open-ended questions. The outline of the sections is as follows:

1. Eligibility: understanding of the study, consent, age, country of professional experience, experience with older adults with dysphagia.
2. Sociodemographic information: clinical experience (years), profession, setting, sex, ethnic background, faith.
3. Experiences of EDAR in older adults: confidence in decision-making, ratio of work related with it, the appropriate way to undertake it, education/training, current role in decision-making, process and participants of the decision-making, likeliness to support an EDAR decision, benefits and challenges, managing different views amongst patients and families, risk management, available support, key factors in the decision-making.
4. Future hopes in EDAR in older adults: what support would be beneficial (education, framework, specialist input, support within the workplace, continuity of care), any other beneficial support methods, any other comments on the topic.

There was a total of 32 questions. Participants were required to answer all questions except for the two final open-ended questions.

### Statistical analyses

We analysed data using R (version 4.2.0) (R Studio Team, 2020). The dependent variables in the models were Confidence, Support Likeliness (likeliness to support an EDAR decision) (0 to 10), Beneficial (how beneficial they find EDAR to be) (0 to 10), and Difficulty (how difficult they find EDAR decision-making to be) (0 to 10), measured on a continuous scale. The predictor variables included Country (UK or Japan), Experience (years), Profession, Setting, Sex, Training, Role in Decision-Making, and Rate of Clinical Work Relating to EDAR. Ordinary least squares (OLS) regression models were fitted for each outcome. Due to evidence of heteroscedasticity (as indicated by Breusch–Pagan tests), robust standard errors were estimated. As a sensitivity analysis, robust regression models were also calculated.

## Results

A total of 1452 responses were obtained, including 1058 from Japan and 394 from the UK. Participant demographics are shown in Table 1. Participants from Japan were older, had more years of clinical experience, and were predominantly doctors, whilst participants from the UK were mostly speech and language therapists (SLTs).

**Table 1:** Demographics of participants

	Japan (*n* = 1058)	UK (*n* = 394)
**Age (median, IQR)**	46 (16.8)	37 (19)
**Sex (Male; *n* %)**	515 (48.7%)	56 (14.2%)
**Occupation**		
Doctor	478 (45.2%)	76 (19.3%)
Nurse/Nurse assistant	167 (15.8%)	19 (4.8%)
Specialist nurse/APN	21 (2.0%)	23 (5.8%)
Physician assistant	0 (0%)	1 (0.3%)
Advanced care practitioner	1 (0.1%)	4 (1.0%)
SLT	145 (13.7%)	245 (62.2%)
Physiotherapist	28 (2.6%)	4 (1.0%)
Occupational therapist	12 (1.1%)	3 (0.8%)
Pharmacist	43 (4.1%)	0 (0%)
Dietician	50 (4.7%)	8 (2.0%)
Dentist	55 (5.2%)	1 (0.3%)
Dental hygienist	16 (1.5%)	0 (0%)
Social worker	8 (0.8%)	0 (0%)
Caregiver	28 (2.6%)	10 (2.5%)
Administrator	3 (0.3%)	0 (0%)
Other	3 (0.3%)	0 (0%)
**Setting**		
Acute hospital	520 (49.2%)	222 (56.3%)
Non-acute hospital	201 (19.0%)	26 (6.6%)
Out-patient clinic	69 (6.5%)	13 (3.3%)
Care home/nursing home	93 (8.8%)	19 (4.8%)
Hospice	13 (1.2%)	9 (2.3%)
Community/Home-based care	148 (14.0%)	102 (25.9%)
Other	14 (1.3%)	3 (0.8%)
**Clinical experience (years; median, IQR)**	16 (14)	10 (14.8)
**Ethnic background**		
White	26 (2.5%)	316 (80.2%)
Asian	1023 (96.7%)	41 (10.4%)
Black	0 (0%)	14 (3.6%)
Hispanic	1 (0.1%)	2 (0.5%)
Other	8 (0.8%)	21 (5.3%)
**Faith**		
Christian	33 (3.1%)	142 (36.0%)
Muslim	0 (0%)	8 (2.0%)
Hindu	1 (0.1%)	9 (2.3%)
Buddhist	420 (39.7%)	6 (1.5%)
Jewish	1 (0.1%)	6 (1.5%)
Other	43 (4.1%)	19 (4.8%)
None	560 (52.9%)	204 (51.8%)

(IQR: interquartile range, APN: advanced practise nurse, SLT: Speech and language therapist)

### Experiences of applying Eating and Drinking with Acknowledged Risks by healthcare professionals

#### Confidence in decision-making

**Figure 1:**
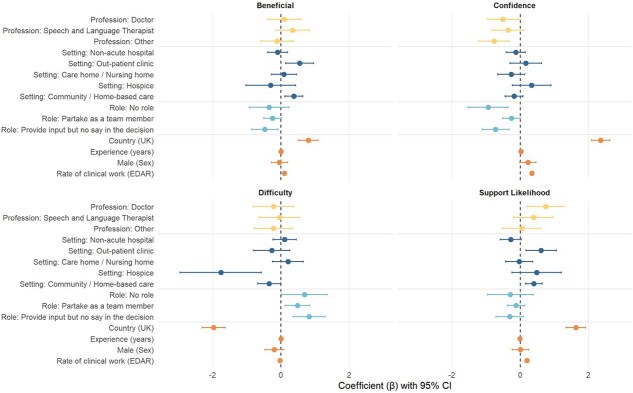
Forest Plots of each factor by outcome.

When a patient shows preference to continue eating and drinking despite risks of aspiration or choking, healthcare professionals assess whether EDAR is appropriate or other methods should be sought, such as modified diets or NBM. Confidence towards EDAR decision-making was reported on a scale of 0 to 10 (10 being the highest confidence) in the survey. Figure 1 and Supplementary Appendix 3 presents the OLS regression results with robust standard errors. UK-based respondents in comparison to Japanese respondents reported significantly higher levels of confidence (β = 2.358, SE = 0.137, *P* < .001), greater likelihood of supporting EDAR (β = 1.633, SE = 0.148, *P* < .001), more likeliness to acknowledge perceived benefits in EDAR (β = 0.804, SE = 0.147, *P* < .001) and lower levels of difficulty in undertaking EDAR (β = −1.970, SE = 0.173, *P* < .001).

#### Factors to support confidence

**Greater years of experience** was associated with higher confidence (β = 0.028 per year, SE = 0.005, *P* < .001). Rate of clinical work related to EDAR was a significant positive predictor for confidence (β = 0.341, SE = 0.022, *P* < .001), likelihood of support (β = 0.203, SE = 0.024, *P* < .001), and perceived benefits of EDAR (β = 0.104, SE = 0.022, *P* < .001) suggesting that a greater rate of clinical work with EDAR was associated with higher confidence, greater likelihood of support for EDAR and greater acknowledgement of its perceived benefits.

**The role in decision making** also significantly influenced confidence. For example, compared to the reference category (where participants indicated full decision-making responsibility), respondents with no role in decision-making had lower confidence (β = −0.936, SE = 0.300, *P* < .001) as did those who only provided input to decision making (β = −0.718, SE = 0.201, *P* < .001). Role also had a significant impact on perceived benefits and difficulty, with participants in these categories reporting fewer perceived benefits and higher perceived difficulty with EDAR.

In terms of **profession**, doctors were less likely to report confidence related to EDAR (β = −0.499, SE = 0.229, *P* < .05) compared to other professions. Whether a participant had received training was also a significant predictor, with participants who had received training more likely to report greater confidence (β = 0.630, SE = 0.110, *P* < .001).

**Setting** was also influential towards perceived confidence. Participants working in community/home settings were more likely to report greater support for EDAR (β = 0.400, SE = 0.124, *P* < .001), perceive it as beneficial (β = 0.383, SE = 0.134, *P* < .001) and find it less difficult (β = −0.343, SE = 0.171, *P* < .05), compared to those in acute hospitals. Similarly, those working in out-patient clinics were also were more likely to report greater support for EDAR (β = 0.617, SE = 0.226, *P* < .001) and perceive it as beneficial (β = 0.548, SE = 0.209, *P* < .001). Participants working in hospices were less likely to find EDAR difficult in comparison to those working in acute hospitals (β = −1.758, SE = 0.606, *P* < .001).

Robust regression analyses produced estimates similar to those from the OLS models, confirming the stability of the findings (Appendix 3).

### Future support for Eating and Drinking with Acknowledged Risks in older adults

**Figure 2:**
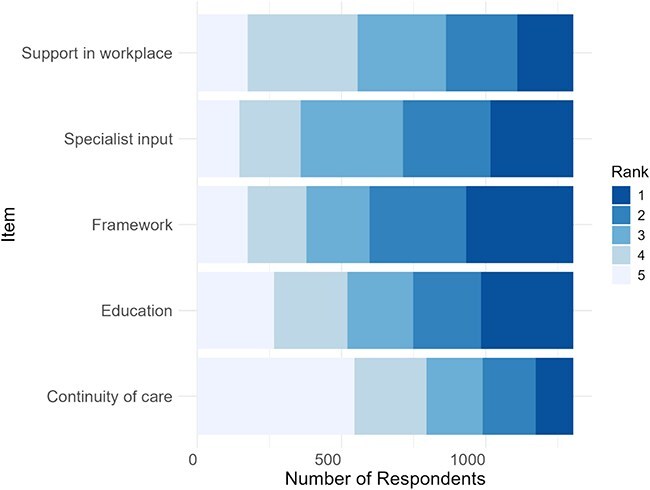
Ranking of what support would be beneficial towards EDAR decision-making.

The results of how participants ranked what support would be beneficial towards EDAR-related decision-making are shown in Figure 2. Framework (guidelines, protocols, legislations, evidence, consent forms/handouts) was ranked to be the most beneficial in both countries. This was followed by education (undergraduate, on the job, conferences, database of cases, increasing awareness), specialist input (multidisciplinary teams, consultation system, swallow assessment availability, facilitating discussions with patients/families, legal backup), support within the workplace (more involvement of your profession in the decision-making, more time to spend on each patient, supervision, peer support in talking with patients/families, opportunity to share case-based experiences) and continuity of care (communication with the community and other hospitals, unified level of care amongst facilities, shared record of previous discussions), in this order.

## Discussion

Our survey distributed amongst healthcare professionals in Japan and the UK revealed differing levels of confidence in EDAR decision-making in both countries. Factors that correlated with confidence were being based in the UK, having longer experience, a greater rate of clinical work related with EDAR, having a role in full decision-making responsibility, having received training in EDAR and being a clinician that is not a doctor (such as an SLT). Existing guidance for EDAR in the UK [5] and sufficient exposure to different situations that can occur with EDAR are understandably beneficial towards confidence. Being a doctor was related with less confidence in EDAR, which may be due to several factors such as having less education, training and exposure to EDAR-related issues, lower capability to work in a multidisciplinary team, and a deeper sense of responsibility for the overall management of patients.

Cultural differences might also play a critical role. Whilst the UK and Japan have similarities in demographics and healthcare systems, there are fundamental differences in healthcare communication, patient empowerment particularly in the older population [22], and advance care planning [23–25], with studies reporting psychocultural-social tendencies affecting ideal shared decision-making. As EDAR in older adults is a culturally and ethically complex decision, there is a need for a culturally-adapted guidance, as suggested in a study on advance care planning [26]. There is also a difference in end-of-life care, where in Japan, a large number of older patients still die in hospitals (64.4% in 2017) [27] in comparison to the UK (46.0%) [28]. The infrastructure for end-of-life care in both countries is still in progress. Different factors including social, cultural (views on ageing and disability), paternalism within the healthcare context (particularly seen in Japan), are all possible factors that may have contributed to the differences observed. To enable EDAR as an option to all appropriate patients, work is needed to increase confidence in EDAR decision-making.

**Figure 3:**
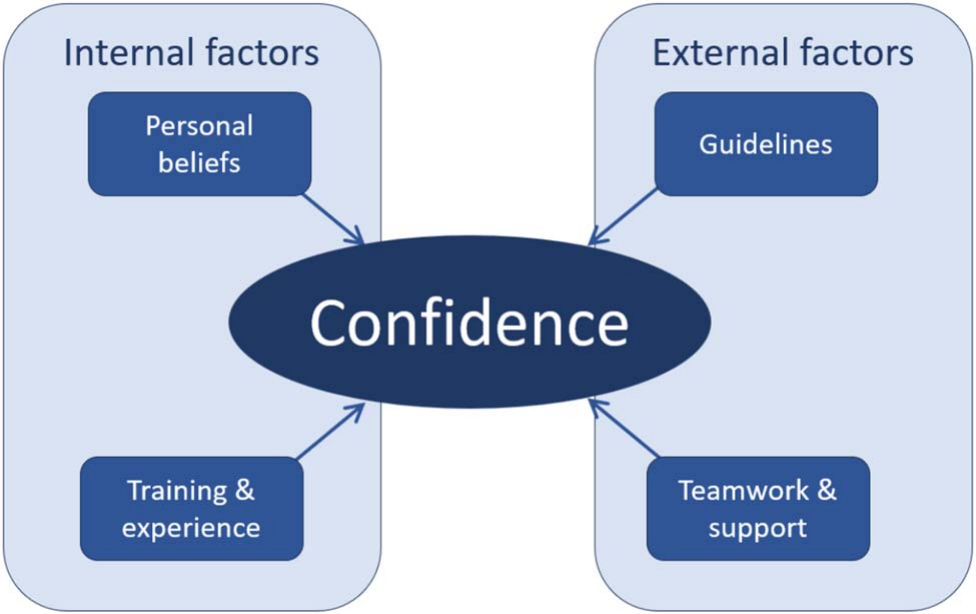
Factors that shape confidence.

This study led to a consideration of what interventions would increase confidence with health practitioners, particularly in Japan. Professionals in both countries ranked ‘Framework’ (consisting of guidelines, protocols, legislations, evidence, consent forms/handouts) as the most beneficial support, followed by education (described as undergraduate, on the job, conferences, database of cases, increasing awareness). This is understandable and consistent with our previous studies highlighting the gap between the need for education on dysphagia/aspiration pneumonia and the lack of an organised curriculum [6, 29, 30]. Whilst guidelines appear to be beneficial, as previously reported in the qualitative phase [6], it will be the other interventions that support the clinicians’ experience, training (internal confidence development) and the support the environment offers (external confidence development). We have illustrated this in a tentative model (Figure 3), which shows particularly promising areas to target in future studies/interventions and demonstrates room for further addition such as patient factors.

Guidance and protocols are interventions to increase confidence by offering external support, yet with a complex pathway as EDAR it will be challenging to keep guidance succinct and relevant. Training and education are reported to be effective in increasing confidence [16]. In particular, the use of simulation is likely to increase confidence [31] as this is closer aligned to real-world situations whilst being in a safe learning environment.

Working with experienced peers and gaining experience through simulation training is another external intervention to increase confidence in healthcare professionals [32]. Especially as EDAR involves a larger multi-disciplinary team in joint decision-making, it is helpful to nurture each team member’s experiences by discussing and sharing the rationale and experiences of EDAR decisions, through actual cases and simulations.

These findings have several clinical and academic implications. Clinically, acknowledging factors that lead to less confidence or more difficulty in decision-making allows us to identify potentially risky or burdensome areas in practise and provide support in an efficient manner, leading to improved experiences in patients, families and healthcare professionals. Multidimensional support via the development of national guidelines and local protocols, multidisciplinary involvement in the decision-making, and support tailored to the individual work setting would be beneficial.

Academically, this study raises further questions towards effective ways of supporting EDAR-related decision-making. This data is also valuable in shaping our education and training of healthcare professionals to ensure relevance to current clinical practise. As raised in the qualitative phase [6] and survey, medical education tends to focus on curative medicine, whilst in practise, end-of-life care and difficult decision-making around this phase of care holds a large part of our caseload.

There are some limitations in this study. This was a survey with predominantly multiple-choice questions. There are biases as with any survey study, including self-selection bias, non-response bias, acquiescence bias and social desirability bias. The results may not be generalisable to other regions. However, this is the first study to explore the experiences of healthcare professionals regarding this complex decision-making process, with a large number of multidisciplinary participants from both countries. Questions and options were developed based on a qualitative study to minimalise bias, and participants were given the opportunity to provide free-form answers. The findings are of value in planning relevant support for each profession, setting and region.

## Conclusion

This study explored the barriers, facilitators, and factors that shape confidence in healthcare professionals for EDAR decision-making in older adults. Confidence was shaped by multiple internal and external factors. Culturally-adapted interventions are needed to enable EDAR for appropriate older adults.

## Supplementary Material

aa-25-2402-File002_afaf380

aa-25-2402-File003_afaf380

aa-25-2402-File004_afaf380

## Data Availability

All data are applicable in the paper.
