# Supplementary material for: Healthcare professionals’ experiences and thoughts on eating and drinking with acknowledged risks in older adults: a comparison of Japan and the UK
Source: Age Ageing. 2026 Jan 22;55(1):afaf380. doi: 10.1093/ageing/afaf380 (PMC12825297; doi:10.1093/ageing/afaf380)
Supplement: aa-25-2402-File003_afaf380 [file aa-25-2402-file003_afaf380.docx]

Healthcare professionals’ experiences and thoughts on Eating and Drinking with Acknowledged Risks in older adults – a comparison of Japan and the UK

Appendix 2. Survey

**Eating and Drinking with Acknowledged Risks in Older Adults: A Comparison of Japan and the UK**

**Invitation** We invite you to take part in our study on decision making for ‘eating and drinking with acknowledged risks (EDAR)’ in older adults. Eating and drinking is a basic right for any person, and an important enjoyment in life. It is common, however, for older adults to be restricted with food and drinks when there is a presumed risk of aspiration. This is common practice, despite evidence that dysphagia is not the sole factor that causes aspiration pneumonia; rather pneumonia is the result of general deterioration related with factors such as comorbidities, age, and malnutrition. Simply restricting patients from eating and drinking is not the answer to preventing aspiration pneumonia. EDAR is an alternative decision that enables comfort, dignity, and autonomy for patients who prefer to continue oral intake despite risks of aspiration/choking.

We understand that decision making around EDAR is complex, with uncertainties around swallowing function, differences in values, and ethical dilemmas. We are conducting this study to investigate the barriers and facilitators to EDAR, and what could be done to better enable EDAR for our older patients.

Initially, comparison of Japan and the UK, which are two similarly ageing societies but with differing cultural, religious and healthcare backgrounds, is expected to enable us to better understand the barriers and facilitators unique to each country or common to both countries, and guide us in the next steps to investigate this topic further.

Ethical approval has been provided by the Health Research Authority (HRA) and Health and Care Research Wales (HCRW) (ID: 321158).

**Eligibility**

Any healthcare professional or care home staff in the UK or Japan who is aged 18 years or above and has the experience of caring for an older patient ( ≥ 65 years old) with difficulty in swallowing /dysphagia or aspiration is eligible to participate.

**Participation requirements**

This study takes no more than 15 minutes. You will be asked to complete a questionnaire about your experiences as a clinician in caring for older adults. Please complete all questions, as your responses will be very valuable.

**Are there any risks or benefits in taking part?**

This study should cause you no harm. Your participation is voluntary and you have the right to stop participating at any time. Your anonymised data will be processed only for the purpose of this study and will not be used for any alternative purpose. Your data will not be used for marketing or commercial purposes. Your answers in this study are confidential, and there are no right or wrong answers. No traceable data will be collected. Data will be stored for 5 years in a secure place. You can withdraw from the study until you submit the final answers. By participating in this study, you will be contributing to future patient care, healthcare professional education and training, and the society in general.

**Contact details**

Yuki Yoshimatsu (yuki.yoshimatsu@nhs.net), David G Smithard (david.smithard@nhs.net)

Elderly Care, Queen Elizabeth Hospital, Lewisham and Greenwich NHS Trust

Stadium Rd, London

SE18 4QH, United Kingdom

First, please answer some questions to check your eligibility.

Q1 I agree to take part in the study described here. I have read and understood the explanation. I understand that my data will be used solely for the purpose of the stated research and its publication.

- Yes (1)
- No (2)

Q2 I understand that my participation is entirely voluntary, that I can choose not to participate, and that I can withdraw before submitting my answers without having to give a reason and without being penalised in any way.

- Yes (1)
- No (2)

Q3 I am a healthcare professional in the following country.

- United Kingdom (1)
- Japan (2)
- Neither (3)

Q4 I have had the experience of caring for an older patient (≥ 65 years old) with dysphagia or aspiration.

- Yes (1)
- No (2)

Q5 What is your age? (Please enter a number)

________________________________________________________________

End of Block: Consent

Start of Block: Demographics

Next, please answer some questions about yourself.

Q6 How many years have you worked in the clinical setting in total? (Please enter a number)

________________________________________________________________

Q7 What is your profession?

- Doctor (1)
- Nurse / Nurse assistant (2)
- Specialist Nurse / Advance practice nurse (3)
- Physician assistant (4)
- Advanced care practitioner (5)
- Speech and Language Therapist (6)
- Physiotherapist (7)
- Occupational Therapist (8)
- Pharmacist (9)
- Dietician / registered dietician / nutritionist (10)
- Dentist (11)
- Dental Hygienist (12)
- Social Worker (13)
- Caregiver (14)
- Administrator (15)
- Other (specify) (16) __________________________________________________

Q8 Which setting is your main place of work?

- Acute hospital (1)
- Non-acute hospital (2)
- Out-patient clinic (3)
- Care home / Nursing home (4)
- Hospice (5)
- Community / Home-based care (6)
- Other (specify) (7) __________________________________________________

Q9 What is your sex?

- Male (1)
- Female (2)

Q10 What is your ethnic background?

- White (1)
- Asian (2)
- Black (3)
- Hispanic (4)
- Other (5)

Q11 What is your faith?

- Christian (1)
- Islam (2)
- Hindu (3)
- Buddhist (4)
- Judaism (5)
- Other (6)
- None (7)

End of Block: Demographics

Start of Block: Experiences in EDAR

Please answer the following questions regarding your experiences in **Eating and drinking with acknowledged risks (EDAR) in older adults**. It may be called ‘**risk feeding**’ or ‘**comfort feeding**’ depending on your work environment.

Q12 How **confident** are you in contributing to EDAR decision making?

|  | Not confident | Very confident |
| --- | --- | --- |

|  | 0 | 1 | 2 | 3 | 4 | 5 | 6 | 7 | 8 | 9 | 10 |
| --- | --- | --- | --- | --- | --- | --- | --- | --- | --- | --- | --- |

| () | 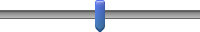 |
| --- | --- |

Q13 How much of your clinical work relates with EDAR decision making?

|  | Few cases | Most cases |
| --- | --- | --- |

|  | 0 | 1 | 2 | 3 | 4 | 5 | 6 | 7 | 8 | 9 | 10 |
| --- | --- | --- | --- | --- | --- | --- | --- | --- | --- | --- | --- |

| () | 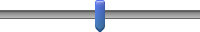 |
| --- | --- |

Q14 What do you feel is the **appropriate** way to undertake EDAR in older adults?

- Allow patient to eat and drink as they wish (1)
- Allow patient to eat and drink but avoid high risk foods (2)
- Allow intake of minimal foods to ensure safety (3)
- EDAR should not be allowed (4)
- I do not know (5)
- Other (specify) (6) __________________________________________________

Q15 Have you had any **education or training** regarding decision making in EDAR?

- Yes (1)
- No (2)

Skip To: Q17 If Have you had any education or training regarding decision making in EDAR? = No

Q16 What training have you had regarding decision making in eating and drinking? (select all that apply)

- Lectures during undergraduate training (1)
- Simulations during undergraduate training (2)
- Lectures at workplace (3)
- Simulations at workplace (4)
- Voluntary participation in seminars outside of work (5)

Q17 What is your **current role** in decision making for eating/drinking?

- Main decision maker (1)
- Partake as a team member (2)
- Provide input but no say in the decision (3)
- No role (4)

Q18 Who participates in the **decision making** for eating and drinking in older adults in your setting? **(select all that apply)**

- Patient (1)
- Family (2)
- Doctor (3)
- Nurse / Nurse assistant (4)
- Specialist Nurse / Advance practice nurse (5)
- Physician assistant (6)
- Advanced care practitioner (7)
- Speech and Language Therapist (8)
- Physiotherapist (9)
- Occupational Therapist (10)
- Pharmacist (11)
- Dietician / registered dietician / nutritionist (12)
- Dentist (13)
- Dental Hygienist (14)
- Social Worker (15)
- Caregiver (16)
- Administrator (17)
- Other (specify) (18) __________________________________________________
- Not sure (19)

Q19 What **process** do you always follow when considering EDAR in older adults? **(select all that apply)**

- National guideline (1)
- Local protocol (2)
- Discuss with designated team or specialist (3)
- Discuss with non-specialist colleagues (4)
- Follow own original protocol (5)
- No set process (6)
- Not applicable (7)

Q20 If an older patient or their family prefers EDAR, how likely are you to **support** this decision?

|  | Very unlikely | Very likely |
| --- | --- | --- |

|  | 0 | 1 | 2 | 3 | 4 | 5 | 6 | 7 | 8 | 9 | 10 |
| --- | --- | --- | --- | --- | --- | --- | --- | --- | --- | --- | --- |

| () | 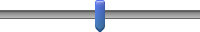 |
| --- | --- |

Q21 In older patients who wish to eat and drink, how **beneficial** do you think EDAR is?

|  | Not beneficial | Very beneficial |
| --- | --- | --- |

|  | 0 | 1 | 2 | 3 | 4 | 5 | 6 | 7 | 8 | 9 | 10 |
| --- | --- | --- | --- | --- | --- | --- | --- | --- | --- | --- | --- |

| () | 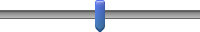 |
| --- | --- |

Q22 What do you think the **benefits** of EDAR are in older adults? **(select all that apply)**

- Patient comfort / quality of life (QoL) (1)
- Family satisfaction (2)
- Staff satisfaction (3)
- Nutrition (4)
- Hydration (5)
- Oral hygiene (6)
- De-medicalisation of end-of-life care (7)
- Other (specify) (8) __________________________________________________
- No benefit (9)

Q23 How **difficult** do you feel it is to make decisions regarding EDAR in older adults?

|  | Not difficult | Very difficult |
| --- | --- | --- |

|  | 0 | 1 | 2 | 3 | 4 | 5 | 6 | 7 | 8 | 9 | 10 |
| --- | --- | --- | --- | --- | --- | --- | --- | --- | --- | --- | --- |

| () | 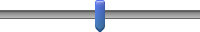 |
| --- | --- |

Q24 What **challenges** have you experienced with the decision-making process for EDAR in older adults? **(select all that apply)**

- Patient safety concerns (1)
- Family's wishes differing from patient's wishes (2)
- Lack of patient's mental capacity (3)
- Legal concerns (4)
- Lack of national guidelines (5)
- Lack of local protocols (6)
- Lack of specialist input (7)
- Lack of knowledge (8)
- Lack of experience (9)
- Lack of continuity of care (10)
- Lack of time to spend on each patient (11)
- Differences in hospital and community (12)
- Other (specify) (13) __________________________________________________
- No difficulties (14)

Q25 When an older patient and family members have differing views on eating and drinking and are **unable to reach an agreement**, how would you most likely manage the situation in your current setting?

- Follow the patient’s wishes (1)
- Follow the family’s wishes (2)
- Ask for a second opinion (3)
- I don't know (4)
- Other (specify) (5) __________________________________________________

Q26 How do you think the **risk** should be managed in older adults who prefer EDAR? **(select all that apply)**

- Perform a detailed swallow assessment (videofluoroscopy or videoendoscopy) (1)
- Minimise risks by allowing only limited foods (2)
- Allow EDAR at home but not in hospital (3)
- Ask families to sign a consent form (4)
- Ask the patient to sign a consent form (5)
- Only agree for EDAR if both patient and family are in agreement (6)
- Consult a specialist (7)
- Make a decision as a multidisciplinary team (8)
- Other (specify) (9) __________________________________________________

Q27 What support is **currently available** to you in making decisions regarding EDAR? **(select all that apply)**

- Colleagues of the same profession (1)
- Colleagues of other professions (2)
- Specialist multidisciplinary team (3)
- National guidelines (4)
- Local protocols (5)
- Training (6)
- Counselling (7)
- Legal backup (8)
- Other (specify) (9) __________________________________________________
- No support (10)
- Not applicable (11)

| 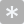 |
| --- |

Q28 What do you think are **key factors** to consider when an older patient wants to EDAR? **(Select the 3 most important factors to you)**

- Swallowing function (1)
- Patient's safety (2)
- Patient's comfort (3)
- Patient's prognosis (4)
- Patient's mental capacity (5)
- Patient’s wishes (6)
- Family’s wishes (7)
- Doctor's judgement (8)
- Speech and Language Therapist's judgement (9)
- Nurse's judgement (10)
- Feasibility in the home and community (11)
- Legal responsibilities (12)
- Other (specify) (13) __________________________________________________

End of Block: Experiences in EDAR

Start of Block: Future hopes

In this final section, please tell us what would be helpful to you in the **decision making** for **Eating and drinking with acknowledged risks (EDAR)**.

Q29 What would **help** you in the EDAR decision making process in older adults? **(select all that apply)**

- Undergraduate education (1)
- Training at work (2)
- Conferences (3)
- Database of cases (4)
- Increasing awareness among professionals (5)
- National guidelines (6)
- Local protocol (7)
- Legislations (8)
- Scientific evidence (9)
- Consent forms (10)
- Handouts for patients and families (11)
- Multidisciplinary specialist team (12)
- Consultation system (13)
- Swallow assessment availability (14)
- Specialist support in facilitating discussions with patients and families (15)
- Legal backup (16)
- More involvement of your profession in the decision-making process (17)
- More time to spend with each patient (18)
- Case-based supervision (19)
- Peer support in difficult discussions (20)
- Opportunity to share case-based experiences (21)
- Enhanced communication within the community (22)
- Unified level of care among facilities (23)
- Shared record of previous discussions (24)
- Other (specify) (25) __________________________________________________
- No further support needed (26)

Q30 How beneficial do you think the following would be for the application of EDAR in older adults, if they were available in your setting? **(Please rank in order of importance)**

______ **Education** (undergraduate, on the job, conferences, database of cases, increasing awareness) (1)

______ **Framework** (guidelines, protocols, legislations, evidence, consent forms / handouts) (2)

______ **Specialist input** (multidisciplinary teams, consultation system, swallow assessment availability, facilitating discussions with patients/families, legal backup) (3)

______ **Support within the workplace** (more involvement of your profession in the decision-making, more time to spend on each patient, supervision, peer support in talking with patients/families, opportunity to share case-based experiences) (4)

______ **Continuity of care**  (communication with the community and other hospitals, unified level of care among facilities, shared record of previous discussions) (5)

Q31 Please list anything else that would help you in EDAR decision making. (optional)

________________________________________________________________

Q32 Do you have any additional thoughts or comments you would like to share on the topic of EDAR? (optional)

________________________________________________________________

End of Block: Future hopes
