# Supplementary material for: Healthcare professionals’ experiences and thoughts on eating and drinking with acknowledged risks in older adults: a comparison of Japan and the UK
Source: Age Ageing. 2026 Jan 22;55(1):afaf380. doi: 10.1093/ageing/afaf380 (PMC12825297; doi:10.1093/ageing/afaf380)
Supplement: aa-25-2402-File004_afaf380 [file aa-25-2402-file004_afaf380.docx]

Healthcare professionals’ experiences and thoughts on Eating and Drinking with Acknowledged Risks in older adults – a comparison of Japan and the UK

Appendix 3. Regression results

|  | *Dependent variable:* | | | |
| --- | --- | --- | --- | --- |
|  |  | | | |
|  | Confidence | Support Likelihood | Beneficial | Difficulty |
|  | | | | |
| Country (UK) | **2.358^***^** | **1.633^***^** | **0.804^***^** | **-1.970^***^** |
|  | **(0.137)** | **(0.148)** | **(0.147)** | **(0.173)** |
|  |  |  |  |  |
| Experience (years) | **0.028^***^** | -0.004 | 0.002 | 0.007 |
|  | **(0.005)** | (0.005) | (0.005) | (0.006) |
|  |  |  |  |  |
| Profession: Doctor | **-0.499^**^** | **0.751^***^** | 0.096 | -0.216 |
|  | **(0.229)** | **(0.275)** | (0.239) | (0.300) |
|  |  |  |  |  |
| Profession: Other Profession | **-0.764^***^** | 0.051 | -0.113 | -0.217 |
|  | **(0.229)** | (0.291) | (0.243) | (0.285) |
|  |  |  |  |  |
| Profession: Speech and Language Therapist | -0.352 | -0.352 | 0.395 | 0.344 |
|  | (0.241) | (0.292) | (0.253) | (0.302) |
|  |  |  |  |  |
| Setting: Care Home/Nursing Home | -0.263 | -0.026 | 0.091 | 0.209 |
|  | (0.200) | (0.203) | (0.186) | (0.230) |
|  |  |  |  |  |
| Setting: Community/Home-based care | -0.176 | **0.400^***^** | **0.383^***^** | **-0.343^**^** |
|  | (0.131) | **(0.124)** | **(0.134)** | **(0.171)** |
|  |  |  |  |  |
| Setting: Hospice | 0.339 | 0.489 | -0.298 | **-1.758^***^** |
|  | (0.289) | (0.369) | (0.371) | **(0.606)** |
|  |  |  |  |  |
| Setting: Non-acute Hospital | -0.125 | **-0.270^*^** | -0.095 | 0.110 |
|  | (0.143) | **(0.158)** | (0.148) | (0.175) |
|  |  |  |  |  |
| Setting: Other | -0.057 | -0.058 | -0.422 | 0.053 |
|  | (0.473) | (0.687) | (0.516) | (0.550) |
|  |  |  |  |  |
| Setting: Out-patient Clinic | 0.168 | **0.617^***^** | **0.548^***^** | -0.267 |
|  | (0.236) | **(0.226)** | **(0.209)** | (0.269) |
|  |  |  |  |  |
| Male (Sex) | **0.228^*^** | 0.012 | -0.044 | -0.197 |
|  | **(0.121)** | (0.124) | (0.121) | (0.137) |
|  |  |  |  |  |
| Training (Yes) | **0.630^***^** | **0.235^**^** | 0.069 | 0.062 |
|  | **(0.110)** | **(0.108)** | (0.114) | (0.137) |
|  |  |  |  |  |
| Role: No Role | **-0.936^***^** | -0.284 | -0.345 | **0.690^**^** |
|  | **(0.300)** | (0.343) | (0.295) | **(0.340)** |
|  |  |  |  |  |
| Role: Team Member | **-0.249^*^** | -0.116 | **-0.242^*^** | **0.484^***^** |
|  | **(0.134)** | (0.131) | **(0.133)** | **(0.181)** |
|  |  |  |  |  |
| Role: Input Only | **-0.718^***^** | -0.303 | **-0.469^**^** | **0.826^***^** |
|  | **(0.201)** | (0.207) | **(0.198)** | **(0.238)** |
|  |  |  |  |  |
| Rate of clinical work (EDAR) | **0.341^***^** | **0.203^***^** | **0.104^***^** | -0.028 |
|  | **(0.022)** | **(0.024)** | **(0.022)** | (0.026) |
|  |  |  |  |  |
| Constant | **3.690^***^** | **5.735^***^** | **6.979^***^** | **7.550^***^** |
|  | **(0.324)** | **(0.365)** | **(0.327)** | **(0.393)** |
|  |  |  |  |  |
|  | | | | |
| Observations | 1,312 | 1,312 | 1,312 | 1,312 |
| R^2^ | 0.464 | 0.261 | 0.111 | 0.165 |
| Adjusted R^2^ | 0.457 | 0.252 | 0.099 | 0.154 |
|  | | | | |
| *Note:* | ^*^p<0.1; ^**^p<0.05; ^***^p<0.01 | | | |
